# The Australian Park Life project: Development of a nationally standardised spatial layer and public participatory GIS for greenspace in Australian capital cities

**DOI:** 10.1016/j.mex.2024.102856

**Published:** 2024-07-25

**Authors:** Paula Hooper, Nicole Edwards

**Affiliations:** The Australian Urban Design Research Centre (AUDRC), School of Design, The University of Western Australia, 35 Stirling Highway, Crawley 6059, Western Australia, Australia

**Keywords:** Greenspace, Public open greenspace, Parks, PPGIS, GIS, Public open space, POS, Geographic information systems, Urban planning, Green space, Spatial data, Park use, Standardised national-scale spatial layer of public open green space for Australian cities and a public participatory GIS to measure use of green spaces

## Abstract

This paper introduces the ‘Australian Park Life Project’ and describes a protocol to standardise the capture and collation of public open greenspace spatial data across Australian cities. This method will progress greenspace research allowing for unique coherent national analyses and comparative research across Australia. We also outline the development of the Park Life public participatory geographic information system (PPGIS) to spatially explore what, and how, public open greenspaces are being used by Australian communities. The combination of community crowdsourced spatial data providing location-specific information on the green public open spaces used, in combination with the national spatial layer of greenspace allows for unique analyses exploring the role of greenspace provision and design on use and represents a transformative strategy in shaping public open space policy and strategy.

• A spatial layer of public open greenspace was created for the eight Australian State and Territory capital cities using a standardised data capture and collection method from local government planning schemes and land use data, and listings of managed and demarcated parks and reserves.
• The Park Life public participatory geographic information system (PPGIS) was built to capture spatially referenced information on the use of greenspaces - specifically what spaces are used, and how they are used.

## Graphical abstract

**Figure:**
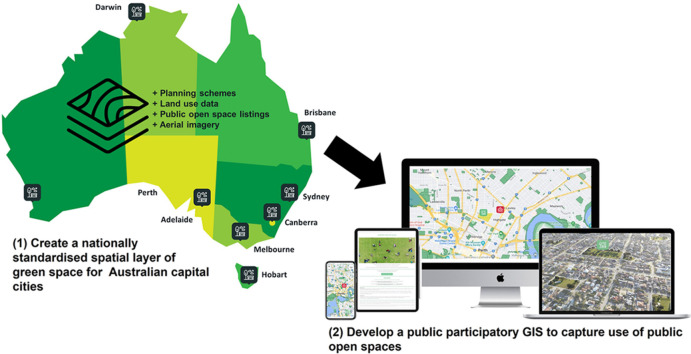

Specifications table

**Table:** 

Subject area:	Environmental Science
More specific subject area:	Urban planning, health promotion
Name of your method:	Standardised national-scale spatial layer of public open green space for Australian cities and a public participatory GIS to measure use of green spaces.
Name and reference of original method:	N/A
Resource availability:	N/A

**Method details**

## Background

In its broadest sense, the term ``public open space'' refers to a variety of publicly available and accessible outdoor areas within urban settings that state and/or local governments have reserved or designated for community (i.e., public) use. In the fields of planning, urban design, landscape architecture, public health, ecology, geography and social sciences, this term encompasses (and if often used interchangeably to describe) a broad spectrum of spaces, including parks, reserves, gardens, sports fields, civic squares, plazas, promenades, streetscapes, greenways, bushland areas, woodlands or forests and nature conservation areas, and the green spaces adjacent to water bodies such river or coastal foreshores [1].

Green public open spaces (hereby referred to as “greenspace”) are defined as areas of land with a predominantly vegetated surface cover and combinations of a variety of natural elements (i.e., the word ‘green’ is used to describe the space as a synonym for vegetation, including natural and planted trees, grass, shrubs, flowers, native bushland or grasses), as a specific subset of the broader spectrum of public open spaces in urban environments. These are typically the focus of public open space strategies and planning policies undertaken by local government authorities on a periodic basis.

Research has shown that interacting with greenspace provides numerous physical and psychological health benefits for urban residents, including reducing stress and anxiety, providing opportunities to experience and interact with nature, space for physical activity and encouraging social interaction and participation, mental restoration, reducing the risk of depression and improving subjective wellbeing [[1], [2], [3], [4]]. However, the variation in health associations with greenspaces observed in the public health and medical sciences literature to date and cross-disciplinary differences may partially stem from differences in how these spaces are measured and the specific greenspaces included [1,5].

### A lack of nationally consistent spatial data of greenspace across Australia

To understand the variation of greenspace across and within Australian states, cities, and local government areas, and to advance robust multi-disciplinary greenspace research, it is essential to be able to perform comparative studies [1]. This necessitates greater alignment and standardisation of greenspace definitions, classifications, terminology, and spatial datasets with a common methodology for measuring greenspaces.

In Australia, the Australian Bureau of Statistics (ABS) meshblock dataset provides a national coverage of public open space information. Meshblocks are the smallest geographical area defined by the ABS, containing between 30 and 60 dwellings. Each meshblock is classified by the ABS according to the dominant land-use – including a ‘parkland’ classification. However, meshblocks are commonly comprised of more than one land use - so whilst the entire meshblock would be classified as “parkland” this may in fact over or under estimate the actual area of parkland depending on the (area of) other land uses that are also present. For example, the meshblock layer Is likely to underestimate smaller parks as by the nature of their size, they were less likely to be classified as the predominant land use within a meshblock. Moreover, the parkland meshblock layer cannot be used to identify the number of individual parks because where multiple open spaces are present in a meshblock these are combined into a single meshblock parkland value. Our previous work has also shown that the types of green spaces classified as ‘parkland’ in the meshblock data do not align well with how greenspace or ‘public open space’ is described in Australian local government public open space planning policies [6]. The results of our previous validation analysis found low intra-class correlation coefficients between computed areas of Meshblock parkland with a WA state-based greenspace dataset that was compiled using local government planning scheme and land use data and listings of managed and demarcated parks and reserves [6].

As such, there is currently no authoritative and nationally consistent, dataset of greenspace across Australia. Existing datasets compiled by different tiers of state and local government are fragmented across jurisdictions, inconsistently released and maintained, and often lack high quality metadata describing their data models, capture techniques and provenance. This has led to a fragmented digital representation both within and between states, as well as on a national level.

The lack of an accurate, standardised digital greenspace dataset hampers abilities to conduct coherent national analyses, monitoring and evaluation, and comparative research across Australian cities and urban areas. Standardised operational definitions, classifications and data capture are needed in order to progress greenspace research and the development of nationally consistent indicators [1].

### A lack of knowledge on the use of public open greenspaces

Internationally, planning policy for public greenspace is typically guided by a standards-based approach that stipulates targets for the provision of a minimal quantity of greenspace per capita or the maximum distances residents should have to travel to access these [[6], [7], [8]]. Whilst this approach is important to ensure equities in the provision of and access to greenspace and the distribution of facilities, amenities, and programming across a community, it fails to consider how community members use the greenspaces [8]. Moreover, few research studies measure the exact greenspaces people use, even though “use” is often hypothesised in the relationships being tested [9]. Measures of visitation to greenspaces are important for understanding patterns of greenspace use in urban settings and its relationship with health behaviours and outcomes – such as physical activity, mental wellbeing and a range of ecosystems services. Knowing which components of greenspace contribute to which benefits will help the future design and managememtn of urban greenspaces to meet the various demands of residents.

The measurement and monitoring of greenspace usage necessitates the use of suitable, reliable, and precise instruments. Traditional methods such as intercept surveys, observational approaches, and momentary time sampling techniques provide data which is limited to the specific greenspaces under study. Consequently, only a few greenspaces within a given area are assessed, making it difficult to generalise findings to identify trends across a community, or in relation to the multiple greenspaces people may frequent [10]. Techniques involving GPS, mobile phone tracking data, accelerometers, or social media 'big data' geolocations and derived information can suffer from inaccuracies and lack sociodemographic representativeness, as not all individuals use social media platforms or are willing to tag themselves at specific locations, or openly share their activities [11]. Furthermore, these methods do not provide insights into the activities performed or the motivations and factors influencing greenspace use [11,12].

Given these limitations, self-report surveys remain a viable option for large-scale studies of greenspace use [12]. Moreover, the shortcomings of existing methods for measuring greenspace use highlight the need for a spatially contextualized self-report approach. This would enable participants to specify the greenspaces they are referring to and describe how they utilize these areas. It is increasingly common for spatial data to be collected from the public to understand people's use and experiences and perceptions of places and spaces using digital public participatory mapping tools referred to as Public Participation Geographic Information Systems (PPGIS). Other terms include volunteered geographical information (VGI), crowd-sourced GIS, geographic citizen science, soft GIS or geo-questionnaires. The web has emerged as a popular platform for PPGIS, enabling surveys to be conducted more rapidly, eliminating the potentially intimidating environment of face-to-face interactions, and allowing individuals to express their opinions fairly and at their convenience [13].

This paper describes the methods of the Australian Park Life Project that was established in direct response to these gaps in the research literature and planning policy and practice for public greenspace provision. The objectives were to:

(1) Develop of a nationally standardised spatial layer of greenspace across Australia.
(2) Develop a public participatory mapping tool to spatially capture the use of greenspaces.

### A standardised definition of public open greenspace

The Australian Park Life Project was focused on publicly accessible greenspace. Our definition of land included in our spatial layer of greenspace was defined from an urban planning perspective based on land tenure and zoning of the land use.

***Publicly accessible greenspace is defined as:*** All areas of public land formally identified, reserved, planned and maintained by local or state governments for the provision of vegetated green-space and natural environments for the purpose of providing recreational and/ or environmental purposes. These spaces are available for use and accessible by the general public with no barrier to entry and free to use. This includes parks, reserves, ovals, playing fields, gardens, bushland, nature reserves and linear or linking spaces.

• ***‘****Public’****refers to the ownership of the space and means:*** Land owned or leased by a national, state or local government bodyand dedicated for community use and the primary purpose of sport, recreation and leisure and/or conservation.
• *‘Open'****refers to the accessibility of the space and means:*** The space is outdoors and accessible for public access and use at any time free of charge. There is no barrier to entry or use by the general public and access to the site is not physically restricted (i.e., fenced off).
• *‘Green’****describes the land surface cover and means:*** Land with a predominantly vegetated surface cover and combinations of a variety of natural (green) elements – including grass (turf), plants / flora, trees and remnant vegetation including all types of native vegetation communities, including forest, woodland, native grasslands, mallee, coastal heathland, or rainforest as well as natural coastal or riparian areas.

## Study area

**Fig. 1:**
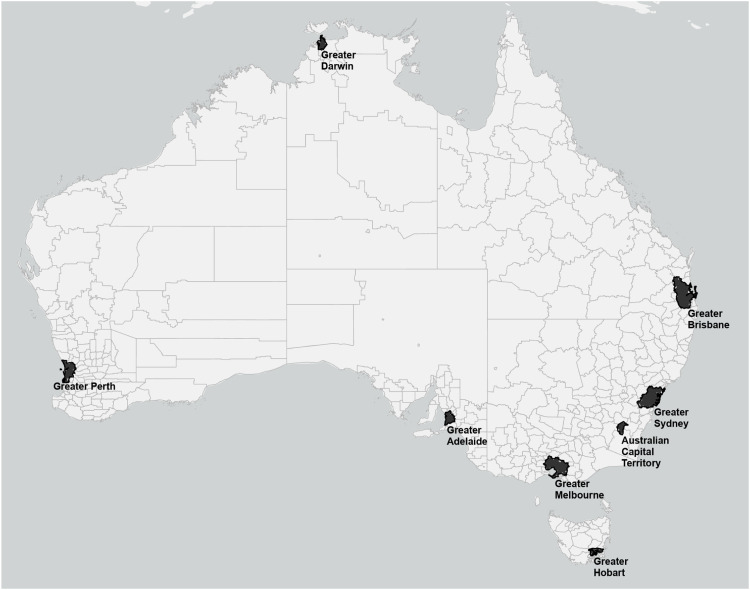
Study extent: the Australian greater capital city statistical areas (■) and local government areas (□).

The focus of this national study was on the metropolitan areas of the eight Australian State and Territory capital city areas (Fig. 1). The spatial extent of the capital cities was defined using the Australian Bureau of Statistics (ABS) Greater Capital City Statistical Areas (GCCSA). The GCCSA reflects the functional extent of each capital city [14]. This extends beyond the built-up edge of the city to include the people who regularly socialise, shop or work within the city, but live in towns and rural areas surrounding the capital city. All local government areas (*n* = 142) that intersected the GCCSA extent were identified for inclusion in the project.

### Part 1: development of a nationally standardised spatial database of greenspace in Australian cities

#### Sourced greenspace data

An extensive search of state and local government resources, along with open data websites, was conducted to identify spatial datasets or planning schemes delineating land designated as 'public open space' within the capital city areas. The spatial datasets identified for each capital city and their respective local government areas (LGAs) are detailed in the supplementary materials section at the end of this article. Additionally, for each local government, listings of parks and reserves found on their websites, as well as those listed in the most recent public open space strategies, were identified and are also outlined in the supplementary materials section.

#### Spatial layer development – a standardised approach to data capture and collation

***Step 1 – base layer:*** All spatial analyses were conducted in ArcGIS Pro (ESRI v3.1). Firstly, an initial base (polygon) spatial layer of publicly accessible greenspaces was created for each capital city by extracting the land areas zoned as “public open space” (or equivalent) for each local government area from the datasets table in the supplementary materials section.

***Step 2 – verification and quality control:*** Next, all parks and reserves listed on local government websites and/or in the public open space strategies were identified and cross-checked with the base spatial layer of greenspaces. Matched spaces (i.e., those present in both the base greenspace spatial layer and the local government parks/reserves listings) were verified and ground-truthed using high-resolution aerial imagery. Any parks or reserves listed on the local government website or open space strategy but not included in the greenspace base layer were verified against the high-resolution imagery and manually digitized. Similarly, any other public open greenspaces (i.e., polygons) included in the base layer but not listed by the local government were visually checked and verified against the aerial imagery. Finally, each local government area was scanned against the aerial imagery, and any additional public open greenspaces identified (that were not in the land zoning database or web listings) were verified and manually digitized.

The final data layer included 55,844 greenspace polygons. Table 1 presents the number of mapped greenspaces captured in each greater capital city area.

**Table 1:** Number of mapped greenspaces by capital city.

Greater Capital City Area	Number of Greenspaces
Adelaide	4704
Brisbane	6011
Canberra	1964
Darwin	469
Hobart	767
Melbourne	21,985
Perth	7288
Sydney	12,656

### Part 2: the park life PPGIS for measuring community use of public open greenspaces

The Australian Park Life Survey was developed as an online geo-questionnaire in which participants individually answered a series of questions, accompanied by interactive maps that provided geographical context and allowed them to spatially reference the parks they use and how they use them. The survey was generated with Angular CLI using Angular Material components, Typescript, Firebase (database), and SASS (CSS / styling). Web maps inside the survey were built from Leaflet and contained Google® base maps and search functionality. The interactive map included the standard functions of panning and zooming. In addition, respondents were able to toggle between the classic Google Maps® roadmap display or satellite view. The cartography associated with the Google® Maps interface is widely recognisable. Therefore, Google® base maps were chosen to provide users with a familiar experience when mapping parks.

The spatial polygon layer of greenspaces captured within the Australian capital city areas were displayed in the mapping window, overlaid on the Google Maps® display to help respondents locate and identify greenspaces. Hovering over, or selecting a polygon, displayed the name of the greenspace. When identifying the greenspaces used, participants could select a greenspace by clicking on a polygon which then marked a pin on the map. The greenspace name (plus unique identifier code), and x,y coordinates of the mapped pin location were recorded. Participants were not restricted to selecting the demarcated greenspace polygons. Any other location (i.e., outside of a greenspace polygon) on the map could be identified by selecting the desired location with a pin and the x,y coordinates were recorded.

The project was stored in AWS S3, and the spatial and survey data was captured and stored in Firebase®. This allowed the research team to easily integrate geographic information systems methods, spatially linked park data, and individual respondent information and characteristics.

#### The Australian Park Life PPGIS protocol

**Fig. 2:**
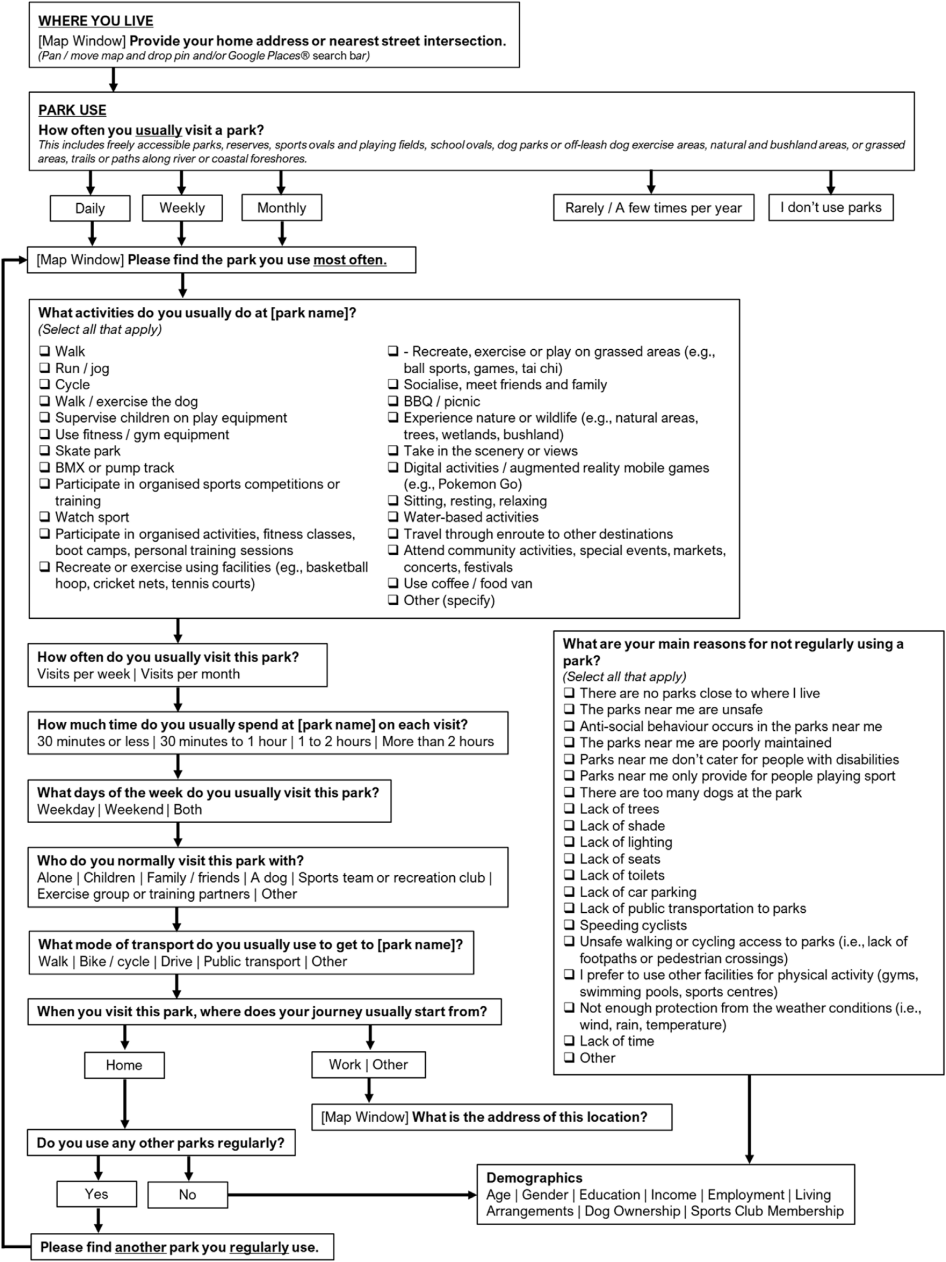
The Park Life PPGIS - protocol.

**Fig. 3:**
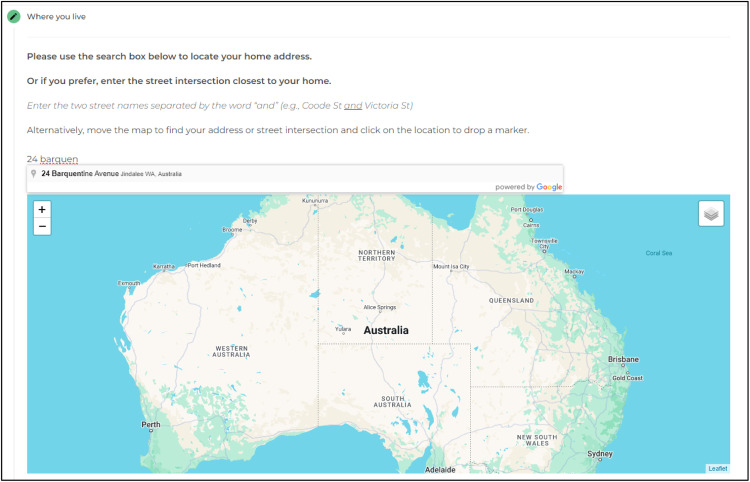
The Park Life PPGIS mapping interface – locating the home address.

**Fig. 4:**
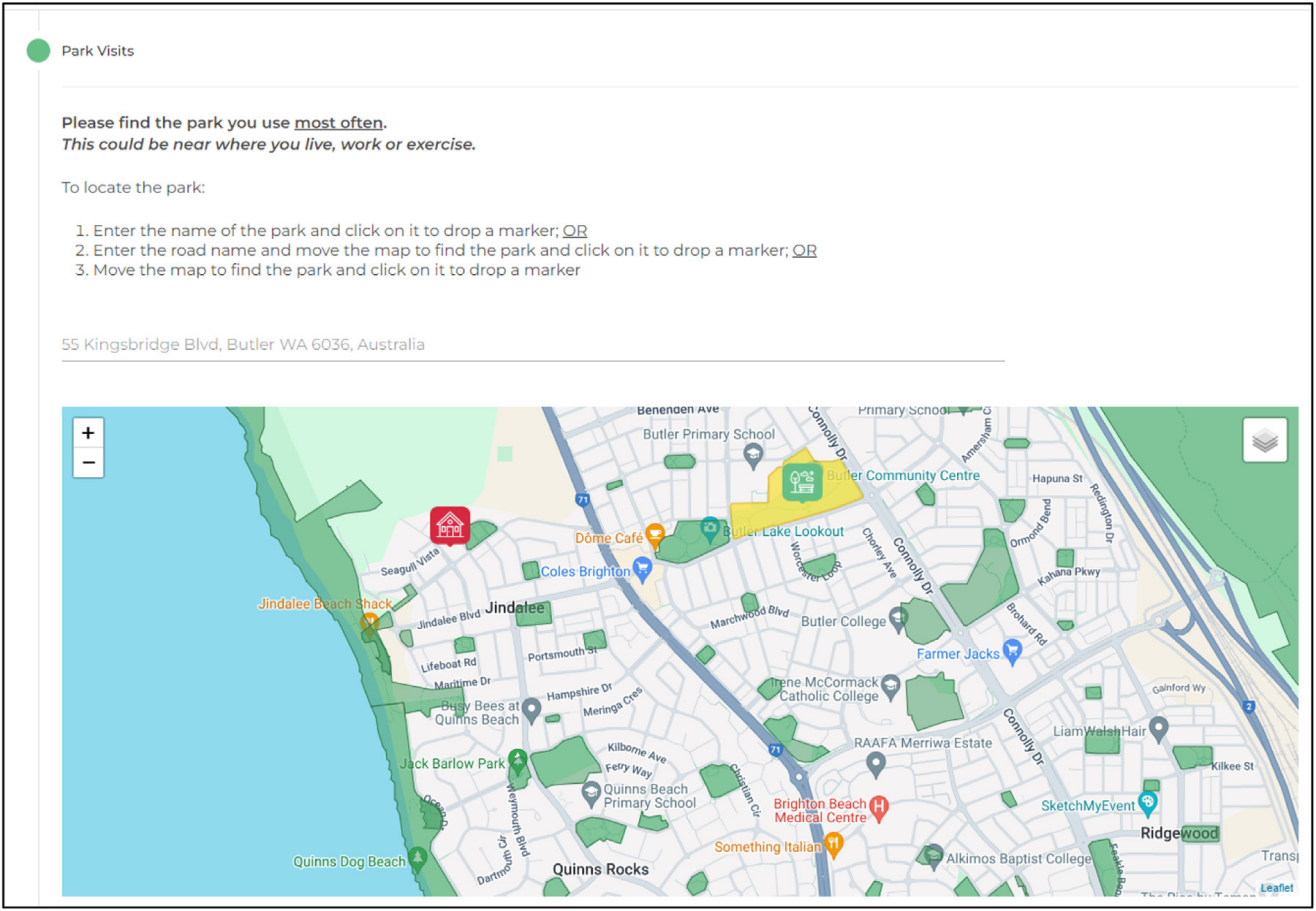
The Park Life PPGIS mapping interface – locating a park (map window zoomed to the spatial extent of the identified home location) and the spatial dataset of greenspace polygons diaplayed.

**Fig. 5:**
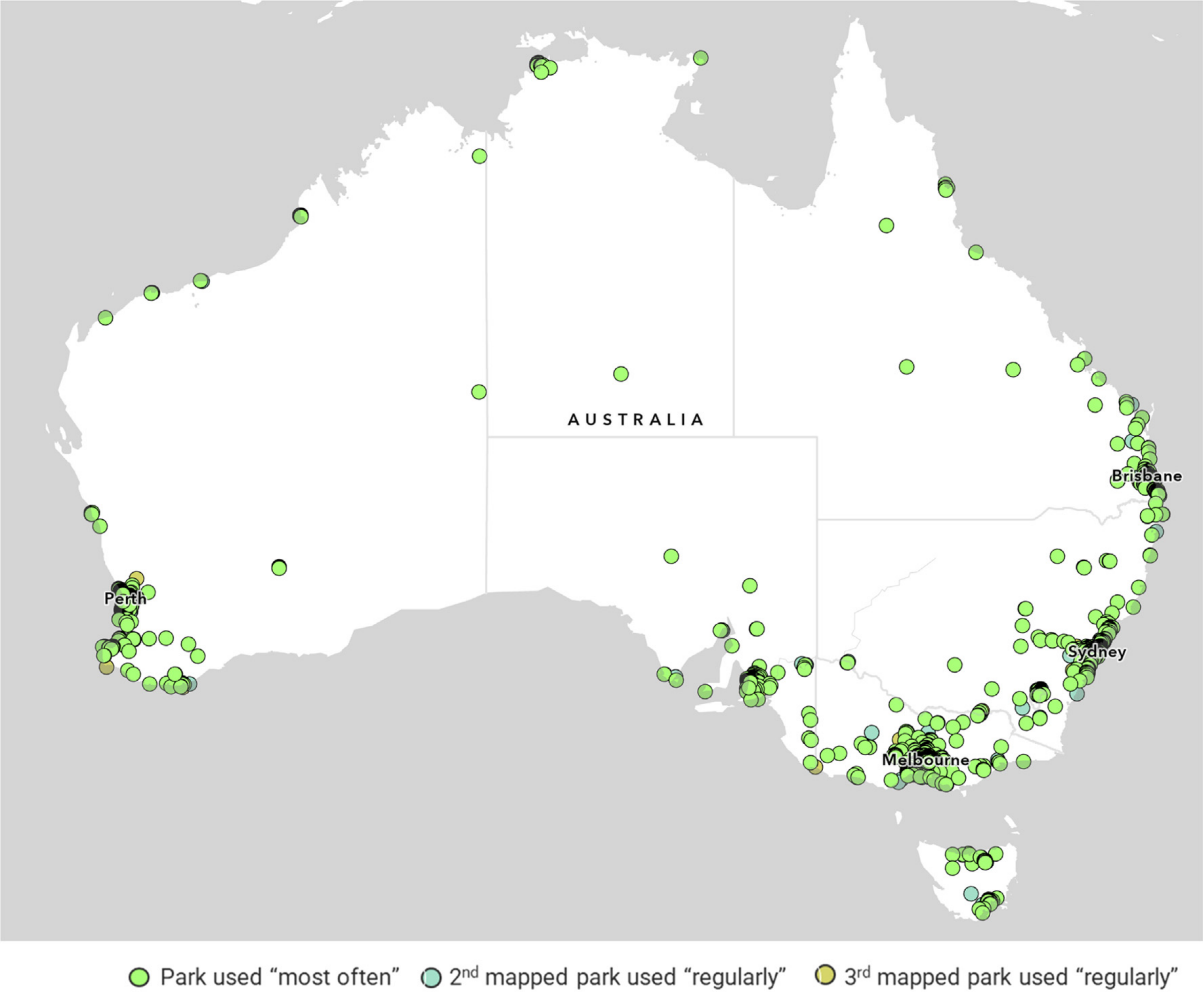
Greenspaces mapped (*n* = 10,026) by Park Life Participants (*n* = 7941) across Australia.

The full Park Life protocol and surveyquestions is outlined in Fig. 2. Screenshots of the mapping interface are shown in Figs. 3 and 4. All questions were informed by previously implemented local government surveys – identified via a review of 165 local government public open space strategies, park and greenspace literature, and a panel of experts comprising local government parks and planning managers, urban planning officers, and planning consultants (Fig. 5).

Participants first identified their home address, or nearest street intersection if they preferred not to provide their full street address. The starting map extent was set Australia-wide and participants could pan and move the map window to find and drop a ‘home’ pin at their address. Alternatively, an address could be entered in the Google Places® search bar, the map would zoom to this location and a target icon would appear for the user to confirm the address by clicking the map which dropped a “home pin”.

The focus of the Australian Park Life survey was concerned with park use in a ‘usual week’ to capture habitual, typical, repeated, regular or ordinarily occurring behaviour concerning park use. A screening question determined if the participant was a ‘park user’ with the question: *“How often do you usually visit a park?”*. Response options were: daily, weekly, monthly, rarely / a few times a year, or ‘I don't use parks. Participants who indicated they used parks' daily’, ‘weekly’ or ‘monthly’ were deemed to be ‘park users’.

The ‘park users' were then instructed to locate *“the park they use most often”* on the map. Again, this could be found by moving the map window to identify and locate the park or entering the park name or street address in the Google Places® search box. The map would zoom to this location and a target icon would appear for the user to confirm the address by clicking the map which dropped a “park pin”.

Following identification of the greenspace, a set of questions specific to use of that greenspace were presented. This included: (1) the activities undertaken; (2) usual frequency of visitation; (3) usual duration of visit; (3) usual day/s of the week the park is visited; (4) who they usually visited the park with; (5) the usual mode of transport to the park; (6) the usual origin of the journey to that park. If they visited the park from a ‘work’ or ‘other’ location another mapping window was displayed, and they were asked to indicate this origin address (or the nearest street Intersection of this location). Again, this could be found by using the Google Places® search bar or locating it directly in the map window. The comprehensive list of 23 park-based activities or recreational experiences participants could choose from covered four broad conceptual categories of activities and anticipated benefits: physical, social, psychological and environmental [2,15].

Next, participants were asked to indicate if they “*normally use any other parks?*” Participants who selected ‘yes’ were asked to “*find another park you regularly use*” in the mapping window and the same set of questions were answered for the identified park. There was no limit on the number of parks participants could add / map.

Participants who indicated they used parks ‘rarely / a few times per year’ or ‘don't use parks’ were directed to a question that prompted them to select reasons why they do not typically use parks. An open-ended question asked them to indicate what would make them use parks.

Social demographic variables often moderate built environment and human health relationships. As such, demographic data typically used for studies that examine associations between the use or availability of greenspaces and health outcomes were collected. Demographic questions captured information on participants' gender, age, education, income, employment situation, living arrangement, dog ownership, sports club/team membership and volunteerism.

#### Reliability of the park life PPGIS

The accuracy of the data collected from participatory GIS methods can be influenced by participants' understanding of the mapping tools and their ability to accurately recall and report their park use. The Park Life PPGIS has been shown to be a reliable instrument to capture greenspace use and activities [9]). Test–retest results on the Park Life PPGIS (*n* = 91 participants) indicate the recall of individual items all showed acceptable (or higher) reliability and confirmed that people can reliably recall details of their greenspace use and associated activities and locate the spaces they use on a digital map [9].

Ground truthing of the test and retest mapped parks (*n* = 204) was conducted to verify that the reported activities in the parks corresponded with the features, facilities, and amenities present within the used greenspaces identified. All mapped greenspace pins outside the demarcated greenspace polygons were assessed and categorised by type (e.g., golf courses, private sports facilities, beaches). Street network distances between the origins (home or other identified address) and the used parks were also computed and compared with the reported modes of transport to the parks. This comparison showed good alignment between the reported transport modes and the calculated travel distances. Specifically, instances where walking and cycling was reported aligned with shorter travel distances, while instances where driving was reported aligned with longer travel distances.

The ground truthing suggested that the mapped parks and associated activities were accurately represented. This robust verification process ensures the reliability of the dataset and enhances confidence in data collected. The Park Life PPGIS demonstrates face validity and enables the reliable capture of greenspace use in large-scale studies. This tool advances the measurement of greenspace use behaviors by explicitly addressing the absence of precise park location data in public health research and public open space planning policy and practice. Moreover, the collection of exact greenspace location data, combined with park-based activity data, provides the accurate information necessary for improved public open space planning.

#### The Australian Park Life PPGIS – deployment and recruitment

The Australian Park Life survey was deployed across Australia for a 12-week period during February to May 2023. A snowball sampling recruitment method was employed, reaching potential participants through various channels aligned with the target population and requesting them to promote or share the survey URL and information with other potential participants [16,17]. Recruitment efforts included:

(1) All local government authorities (*n* = 142) within the capital city regions were invited to participate in the study. An email was sent to the public open space coordinator or planner (or equivalent role) where these could be identified, or to the general / reception email address where respective role or personnel could not be identified. The email invited the local government to participate in the project and to assist with the promotion of the survey to their local community in exchange for an report on the results from their community. A total of 19 local governments across the country participated and assisted with recruitment which included the promotion of the survey through their Facebook and social media pages, on their community consultation webpages,to community groups, and the installation of promotional signs in parks and community facilities.
(2) Emails were sent to *n* = 518 non-governmental organisations, community groups, resident action groups, residents associations, volunteer groups, friends of groups, landcare groups, coastcare groups, environmental groups, community garden groups, men's sheds, walking groups, bushwalking groups, sports clubs, and rotary clubs requesting they promote the survey to their members through the distribution of a flyer promoting the survey (with a QR code) and/or the survey weblink (URL).
(3) Professional associations and industry bodies related to parks and public open space planning, landscape architecture, urban planning were asked to promote the survey to their members via e-newsletters, including the state division of Parks and Leisure Australia, the Planning Institute of Australia, the Urban Development Institute of Australia (UDIA).
(4) Promotion via Facebook community groups – community groups created for residents of suburbs matching the suburbs within the eight capital city metropolitan areas, walking groups, dog owner groups, neighbourhood watch groups, sports groups, and groups for culturally and linguistically diverse communities, were searched on Facebook and requests were made to join the group and post information about and a link to the Park Life survey. A total of 580 Facebook community groups were joined. After an initial post promoting the survey two follow up posts were made at three-week intervals (total of 2320 posts).
(5) Paid advertising through Facebook. In the last four weeks of the survey deployment the paid advertising targeted suburbs within the capital city areas with fewest participants.
(6) Weekly LinkedIn posts promoting the survey were made. Each post used a different tag line prompting people to think about their own park use. The researchers also shared posts relating to the survey made by others.All posts used hashtags (#) to promote content categorization and At Signs (@) to maximize reposting and facilitate communication [18].
  • The Australian Park Life survey … Put your park on the map.
  • Love your local park? Tell us about it? Help us map Australia's park use.
  • What's your favourite dog park?
  • How's your park life? Tell us how you spend time in your favourite parks and open spaces.
  • What do you think about your local park?
  • Map your local park. We want to know what parks and green spaces you use, how you use them and what you think of them?
  • Don't use parks? Tell us why not.
(7) The researchers also emailed colleagues asking them to forward the survey promotional materials to their colleagues and students and to post recruitment information on their personal, school, or business social media sites.

A total of *n* = 7941 participants completed the Park Life survey (Table 2), with a total of 10,026 park pins mapped. *N* = 6784 participants (85.4 %) were regular park users who mapped the park they used ‘most often’. Of these park users, *n* = 2547 participants (37.5 %) mapped a second park they regularly used and *n* = 695 (10.2 %) participants mapped a third park regularly used.

**Table 2:** Number of Park Life participants across Australia.

Location	Number of participants	Percentage of participants
Greater Adelaide	227	2.86 %
Greater Brisbane	253	3.19 %
Canberra *(Australian Capital Territory)*	41	0.52 %
Greater Darwin	28	0.35 %
Greater Hobart	61	0.77 %
Greater Melbourne	1125	14.17 %
Greater Perth	4487	56.50 %
Greater Sydney	636	8.01 %
Other regional areas	1083	13.64 %

## Conclusion

The methods presented here enabled the development of a unique national spatial dataset that standardised the collation of data collected from local government planning schemes, land use data and listings of managed and demarcated parks and reserves and the development of a public participatory GIS that captured community use of greenspaces.

The Australian greenspace spatial layer provides a consistent and comprehensive dataset that researchers can rely upon to conduct national-scale spatial analyses and comparative studies across different regions and states. This is crucial for understanding the impact or greenspace in urban areas on public health, social equity, and environmental sustainability to inform evidence-based decision-making. Furthermore, standardisation improves the transparency and reproducibility of spatial analyses, allowing researchers and policymakers to validate and replicate studies, fostering a robust and credible evidence base for public open space planning and health outcomes. This also reduces redundancy and inconsistencies in data collection and processing, saving time and costs associated with managing multiple disparate datasets.

Maintaining an up-to-date layer of greenspace requires continuous monitoring and data updates. However, the use of local government data that is regularly updated with changes such as new park developments or closures, means this approach, and the national spatial layer, can be efficiently updated ensuring that the data remains current and relevant.

The national ‘Park Life’ study provides a unique snapshot of greenspace use across Australian communities and urban areas. This information is essential for understanding the role of greenspace in promoting physical health, mental well-being, and social cohesion. By identifying the factors that influence park use, researchers can develop evidence-based recommendations to enhance the design and management of these public spaces, ensuring they meet the needs of various communities effectively.

Furthermore, leveraging PPGIS for assessments of public open greenspace use marks a significant advancement for the research, policy and practice of public open space provision. The spatial data obtained from the Park Life PPGIS providing location specific information on the greenspaces used, will be merged with the national spatial layer of public open space to allow for unique analyses exploring the role of park provision and design on park use and represents a transformative strategy in shaping public open space policy and strategy. The use of the Park Life PPGIS to capture park use could be easily integrated into local government public open space community consultation surveys to provide benchmarks of community park use and benefits and evaluate any park upgrades or interventions. The Park Life survey and national spatial layer will be incorporated into a participatory planning platform enabling people to map their greenspace use and see them reflected on a crowd-sourced map of their local government area or city.

## Ethics statements

Ethics approval for the project was granted by The University of Western Australia Human Research Ethics Committee (2022/ET000128).

All participants were required to provide their informed consent to participate in the survey by selecting the “Take the survey now” button in response to the statement: If you are over 18 and agree to participate, please click the button below and complete the questions that follow.

The Facebook Community groups often required you to indicate a reason to join the group. In such instances a request was made to join the group clearly stating the purpose of the Park Life study and the intention to promote the survey (via a post and url link to the survey) to the respective group's members.

## CRediT authorship contribution statement

**Paula Hooper:** Conceptualization, Methodology, Software, Validation, Formal analysis, Investigation, Resources, Data curation, Writing – original draft, Visualization, Supervision, Project administration, Funding acquisition. **Nicole Edwards:** Conceptualization, Methodology, Validation, Formal analysis, Investigation, Resources, Data curation, Writing – review & editing, Supervision, Project administration, Funding acquisition.

## Declaration of competing interest

The authors declare that they have no known competing financial interests or personal relationships that could have appeared to influence the work reported in this paper.

## Data Availability

Data will be made available on request.

## Appendix Supplementary materials

Image, application 1
