# Supplementary material for: The Australian Park Life project: Development of a nationally standardised spatial layer and public participatory GIS for greenspace in Australian capital cities
Source: MethodsX. 2024 Jul 25;13:102856. doi: 10.1016/j.mex.2024.102856 (PMC11337695; doi:10.1016/j.mex.2024.102856)
Supplement: Supplementary file 1 [file mmc1.docx]

**Supplementary material - Planning scheme or land use spatial datasets identified in each capital city**

| **GREATER ADELAIDE** | | | |
| --- | --- | --- | --- |
| **Local Government** | **Spatial dataset/s** | **Public Open Space Strategy** | **LGA park / public open space directory** |
| City of Adelaide | “Park Land Parks”  Provides locations and boundaries of each park within the Adelaide City Council area.  Accessed from Data.gov.au  <https://data.gov.au/dataset/ds-sa-8b6bb491-d9e3-48dc-8230-08a24de1773e/distribution/dist-sa-8dad7404-d7e8-4660-b553-667b33dc0d57/details?q=adelaide%20parks> | Adelaide Park Lands Management Strategy 2015-2025  <https://d31atr86jnqrq2.cloudfront.net/docs/strategy-adelaide-parklands-management.pdf?mtime=20190703124947&focal=none> | Adelaide park lands features directory: <https://www.cityofadelaide.com.au/community/adelaide-park-lands/> |
| City of Port Adelaide Enfield | “SA_LandUseGeneralised2019”  South Australian land use scheme derived from the valuation information and land parcel boundaries.  Land use codes selected:   - Reserve - Recreation - Vacant - Education   Accessed from the South Australian Government Data Directory (Data.SA):  <https://data.sa.gov.au/data/dataset/land-use-generalised> | Open Space Strategy 2021-2026  <https://www.cityofpae.sa.gov.au/__data/assets/pdf_file/0035/786239/PAE-Open-Space-Strategy.pdf> | Playgrounds, Parks & Gardens directory:  <https://www.cityofpae.sa.gov.au/explore/sports-and-recreation/playgrounds-parks-and-gardens> |
| City of Charles Sturt |  | Open Space Strategy 2025  <https://www.charlessturt.sa.gov.au/council/council-documents/plans-and-strategies/open-space-strategy> | Parks, reserves and playgrounds directory:  <https://www.charlessturt.sa.gov.au/community/sports-and-recreation/parks-reserves-and-playgrounds> |
| City of West Torrens |  | Draft Open Space Plan 2021-2026  <https://yoursay.westtorrens.sa.gov.au/open-space-plan> | Parks and playgrounds map:  <https://www.westtorrens.sa.gov.au/Community/Children-youth-and-families/Playgrounds-and-parks> |
| The City of Unley |  | Open Space Asset Management Plan 2020  <https://www.unley.sa.gov.au/files/assets/public/council/about-the-council/plan-asset-management-open-space.pdf> | Parks and playgrounds directory:  <https://www.unley.sa.gov.au/Events-programs-facilities/Facilities-venues/Parks-playgrounds> |
| **Local Government** | **Spatial dataset/s** | **Public Open Space Strategy** | **LGA park / public open space directory** |
| City of Marion |  | Open Space Asset Management Plan 2020-2030  <https://cdn.marion.sa.gov.au/sp/City-of-Marion-Open-Space-Asset-Management-Plan-2020.pdf?mtime=20201029125119&focal=none> | Find parks directory:  <https://www.marion.sa.gov.au/things-to-do/parks-and-playgrounds/find-parks> |
| City of Holdfast Bay |  | Open Space and Public Realm Strategy 2018  <https://www.holdfast.sa.gov.au/assets/general-downloads/Council/Strategy-and-plans/1809-CoHB-Open-Space-Strategy_v11FA_digital.pdf> | Parks, reserves and beaches directory:  <https://www.holdfast.sa.gov.au/discover-our-place/parks-and-reserves> |
| City of Mitcham |  | Open Space Asset Management Plan Summary 2020  <https://www.mitchamcouncil.sa.gov.au/__data/assets/pdf_file/0026/774710/Open-Space-Asset-Management-Plan-Summary.pdf>  Portfolio of open space assets:  <https://mitcham.maps.arcgis.com/apps/MapJournal/index.html?appid=705efd85837246bbbe55c7129f385480> | Parks, Reserves & Playgrounds directory  <https://www.mitchamcouncil.sa.gov.au/discover/sports-and-recreation/parks-reserves-and-playgrounds> |
| The City of Norwood Payneham & St Peters |  | Open Space Strategy  <https://www.npsp.sa.gov.au/directory_documents/0_open_space_strategy> | Parks & Playgrounds directory  <https://www.npsp.sa.gov.au/culture_and_lifestyle/parks_and_playgrounds> |
| Campbeltown City Council |  | Open Space Directions and Strategies Report  <https://www.campbelltown.sa.gov.au/__data/assets/pdf_file/0023/234239/Open-Space-Strategy.pdf> | Parks, Reserves & Ovals directory  <https://www.campbelltown.sa.gov.au/recreation-and-leisure/parks-reserves-and-ovals> |
| City of Prospect |  | Open Space Directions And Strategies Report  <https://www.prospect.sa.gov.au/__data/assets/pdf_file/0016/107602/CoP-Open-Space-Strategy_v7_Endorsed_low-res2.pdf> | Parks and gardens directory  <https://www.prospect.sa.gov.au/community/venues-and-parks/parks-and-reserves> |
| City of Salisbury |  |  | Parks and Facilities directory  <https://www.salisbury.sa.gov.au/Activities/Community_Facilities_and_Parks/Parks_and_Facilities> |
| **Local Government** | **Spatial dataset/s** | **Public Open Space Strategy** | **LGA park / public open space directory** |
| City of Playford |  | Open Space Strategy 2018-2028  <https://cdn.playford.sa.gov.au/general-downloads/Policy-Documents/SC-SP_1810_OpenSpaceStrategy2018-28.pdf?mtime=20181102151252&focal=none%20%20%20/https://cdn.playford.sa.gov.au/general-downloads/Plans-and-Guidelines/SC-SP_1810_OpenSpaceGuidelines2018.pdf?mtime=20181102150755&focal=none> | Parks, Reserves and Playgrounds directory  <https://www.playford.sa.gov.au/explore/venues-and-facilities/parks-reserves-and-playgrounds?display=map> |
| Town of Gawler |  | Open Space, Sport & Recreation Plan 2025  <https://www.gawler.sa.gov.au/__data/assets/pdf_file/0028/217783/town-of-gawler-open-space-sport-recreation-plan-2025.pdf.pdf> | Parks & Playgrounds directory  <https://www.gawler.sa.gov.au/recreation/parks-playgrounds> |
| Town of Walkerville |  | 2020-2025 Open Space Strategy  <https://www.walkerville.sa.gov.au/council/strategic-plans/open-space-strategy> | Parks, Reserves and Ovals directory <https://www.walkerville.sa.gov.au/community/recreation-facilities/parks-reserves-and-ovals> |
| City of Burnside |  | - | Parks directory:  <https://www.burnside.sa.gov.au/Parks> |
| City of Onkaparinga |  | Open Space Strategic Management Plan 2018–23  <https://www.onkaparingacity.com/files/assets/public/strategic-plans/open-space-strategic-management-plan-2018-2023.pdf> | Parks and reserves directory  <https://www.onkaparingacity.com/Around-me/Parks> |
| Tea Tree Gully |  | Open Space Policy  <https://www.teatreegully.sa.gov.au/files/assets/public/council-documents/documents/open_space_policy.pdf> | Parks, playgrounds & ovals directory  <https://www.teatreegully.sa.gov.au/Places-Council-parks-facilities-and-venues/Parks-playgrounds-and-ovals> |
| **GREATER PERTH** | | | |
| **Local Government** | **Spatial dataset/s** | **Public Open Space Strategy** | **LGA park / public open space listings** |
| Greater Perth – for all LGAs | “Local Planning Scheme - Zones and Reserves (DPLH-071)”  The zones and reserves dataset shows the zoning for the whole State where there is a Local Planning Scheme in place. This dataset have been standardised where the zone number (for a particular zone) for one scheme, is the same zone number in another scheme, if they have the same zoning.  Data last updated: 16-06-2021  Accessed from DataWA  <https://catalogue.data.wa.gov.au/dataset/local-planning-scheme-zones-and-reserves-dop-025> | - | - |
| **Local Government** | **Spatial dataset/s** | **Public Open Space Strategy** | **LGA park / public open space listings** |
| City of Armadale | Local Planning Scheme - Zones and Reserves (DPLH-071)  Scheme zone for greenspace =   - Parks and recreation - Public purposes | Parks Facilities Strategy  <https://www.armadale.wa.gov.au/parks-facilities-strategy-0> | Parks, playgrounds and reserves directory  <https://www.armadale.wa.gov.au/parks-playgrounds-reserves> |
| Town of Bassendean | Local Planning Scheme - Zones and Reserves (DPLH-071)  Scheme zone for greenspace =   - Parks and recreation - Public purposes | - | Parks and reserves directory  <https://www.bassendean.wa.gov.au/community/venues-vibrancy/parks-and-reserves.aspx> |
| City of Bayswater | Local Planning Scheme - Zones and Reserves (DPLH-071)  Scheme zone for greenspace =   - Local public open space - Special foreshore development | Play Space Strategy <https://www.bayswater.wa.gov.au/CityOfBayswater/media/Documents/Community/Play-Space-Strategy-Updated-October-2022.pdf> | Parks and gardens directory  <https://www.bayswater.wa.gov.au/environment-and-sustainability/parks-and-gardens/parks> |
| City of Belmont | Local Planning Scheme - Zones and Reserves (DPLH-071)  Scheme zone for greenspace =   - Parks and recreation | Public Open Space Strategy 2022 - 2040  <https://www.belmont.wa.gov.au/docs/ecm/public-open-space-strategy-2022-2040-part-2.pdf> | Parks and playgrounds directory  <https://www.belmont.wa.gov.au/discover/places-to-go/parks-and-playgrounds> |
| City of Cambridge (T) | Local Planning Scheme - Zones and Reserves (DPLH-071)  Scheme zone for greenspace =   - Parks and recreation |  | Parks and Facilities directory  <https://www.cambridge.wa.gov.au/Discover/Parks-Facilities> |
| City of Canning | Local Planning Scheme - Zones and Reserves (DPLH-071)  Scheme zone for greenspace =   - Public open space - Education | - | Parks and reserves directory  <https://www.canning.wa.gov.au/recreation-and-community/parks-and-reserves/> |
| City of Claremont (T) | Local Planning Scheme - Zones and Reserves (DPLH-071)  Scheme zone for greenspace =   - Local reserves - recreation | Public Open Space and Community  Facilities Strategy  <https://www.claremont.wa.gov.au/media/alfpwyi5/11-public-open-space-and-community-facilities-strategy.pdf> | Parks and reserves directory  <https://www.claremont.wa.gov.au/environment/parks/> |
| City of Cockburn | Local Planning Scheme - Zones and Reserves (DPLH-071)  Scheme zone for greenspace =   - Parks and recreation | Public Open Space Strategy 2014 – 2024  <https://www.cockburn.wa.gov.au/getattachment/0679576d-43fd-4308-be57-6213e8741d99/ECM_8330032_v17_Public-Open-Space-Strategy-2014_2024-(5-Year-Review)-docx.aspx> | Parks and reserves directory  <https://www.cockburn.wa.gov.au/Parks-and-Reserves> |
| **Local Government** | **Spatial dataset/s** | **Public Open Space Strategy** | **LGA park / public open space listings** |
| City of Cottesloe (T) | Local Planning Scheme - Zones and Reserves (DPLH-071)  Scheme zone for greenspace =   - Local parks and recreation | Public open space and playground strategy 2019  <https://www.cottesloe.wa.gov.au/documents/1509/public-open-space-strategy-final-nov-2019> | Parks directory  <https://www.cottesloe.wa.gov.au/explore-cottesloe/beaches-parks-and-wilderness/parks> |
| East Fremantle (T) | Local Planning Scheme - Zones and Reserves (DPLH-071)  Scheme zone for greenspace =   - Open space | - | Parks and reserves directory  <https://www.eastfremantle.wa.gov.au/community/public-facilities/parks-and-reserves.aspx> |
| City of Fremantle | Local Planning Scheme - Zones and Reserves (DPLH-071)  Scheme zone for greenspace =   - Open space | City of Fremantle Green Plan 2020  <https://www.fremantle.wa.gov.au/sites/default/files/sharepointdocs/Green%20Plan%202020-C-000476.pdf> | Parks and reserves directory  <https://www.fremantle.wa.gov.au/visit/outdoor-spaces/parks-and-reserves> |
| City of Gosnells | Local Planning Scheme - Zones and Reserves (DPLH-071)  Scheme zone for greenspace =   - Local open space - Watercourses | Public Open Space Strategy  <https://www.gosnells.wa.gov.au/About_our_City/Major_initiatives/Public_Open_Space_Strategy/Public_Open_Space_Strategy_and_suburb_maps> | Parks and outdoor spaces directory  <https://www.gosnells.wa.gov.au/About_our_City/Places_Spaces/Parks_and_Spaces_in_your_area> |
| City of Joondalup | Local Planning Scheme - Zones and Reserves (DPLH-071)  Scheme zone for greenspace =   - Public open space - Environmental conservation reserve - Drainage / waterway | Review of Active Reserves and  Community Facilities (2017) <https://www.joondalup.wa.gov.au/files/councilmeetings/2017/Attach13brf171205.pdf> | Parks and reserves directory  <https://www.joondalup.wa.gov.au/kb/resident/parks-and-reserves> |
| City of Kalamunda | Local Planning Scheme - Zones and Reserves (DPLH-071)  Scheme zone for greenspace =   - Local open space - Residential bushland | Public Open Space Strategy  <https://www.kalamunda.wa.gov.au/building-development/planning/strategies/public-open-space-strategy> | Parks and reserves directory  <https://www.kalamunda.wa.gov.au/community/parks-facilities/parks-reserves> |
| City of Kwinana | ocal Planning Scheme - Zones and Reserves (DPLH-071)  Scheme zone for greenspace =   - Park recreation and drainage | Community Infrastructure Plan 2011-2036  <https://www.kwinana.wa.gov.au/council/documents,-publications-and-forms/publications-and-forms-(all)/plans-and-strategies/2024/community-infrastructure-plan-2011-31> | Sports Clubs, Ovals and Sporting Reserves directory  <https://www.kwinana.wa.gov.au/city-life/facilities-and-public-spaces/sports-clubs,-ovals-and-sporting-reserves> |
| City of Mandurah | Local Planning Scheme - Zones and Reserves (DPLH-071)  Scheme zone for greenspace =   - District recreation - Local recreation | Mandurah Active Recreation Strategy 2015 - 2025  <https://www.mandurah.wa.gov.au/-/media/files/com/downloads/learn/strategies-and-plans/mandurah-active-recreation-strategy.pdf> | - |
| **Local Government** | **Spatial dataset/s** | **Public Open Space Strategy** | **LGA park / public open space listings** |
| City of Melville | Local Planning Scheme - Zones and Reserves (DPLH-071)  Scheme zone for greenspace =   - Public open space | Public Spaces Strategy 2017  <https://www.melvillecity.com.au/our-city/publications-and-forms/urban-planning/city-of-melville-public-spaces-strategy> | Parks and reserves directory  <https://www.melvillecity.com.au/things-to-do/find-parks-and-reserves> |
| Mosman Park (T) | Local Planning Scheme - Zones and Reserves (DPLH-071)  Scheme zone for greenspace =   - Public open space | Public Open Space Strategy  <https://www.mosmanpark.wa.gov.au/wp-content/uploads/2024/04/POS-Strategy-Final.pdf> | Parks and reserves directory  <https://www.mosmanpark.wa.gov.au/play/places-2/places/> |
| Mundaring (S) | Local Planning Scheme - Zones and Reserves (DPLH-071)  Scheme zone for greenspace =   - Recreation | Public Open Space Strategy  <https://www.mundaring.wa.gov.au/ResidentServices/Planning/Documents/Strategies/Public%20Open%20Space%20Strategy.pdf> | Parks and reserves directory  <https://www.mundaring.wa.gov.au/leisure-recreation/playgrounds-and-parks.aspx> |
| Murray (S) | Local Planning Scheme - Zones and Reserves (DPLH-071)  Scheme zone for greenspace =   - Public recreation - Public recreation / conservation | Public Open Space Strategy 2022  <https://www.murray.wa.gov.au/documents/267/public-open-space-strategy> | Places and spaces directory  <https://www.murray.wa.gov.au/places-and-spaces> |
| City of Nedlands | Local Planning Scheme - Zones and Reserves (DPLH-071)  Scheme zone for greenspace =   - Public open space - Educational | Public open space strategy - parks in the city of Nedlands  <https://ehq-production-australia.s3.ap-southeast-2.amazonaws.com/4b7d2b582403544395e8efbd06fc017f888c9ea5/documents/attachments/000/096/606/original/Parks_in_the_City_of_Nedlands.pdf?X-Amz-Algorithm=AWS4-HMAC-SHA256&X-Amz-Credential=AKIA4KKNQAKIOR7VAOP4%2F20240630%2Fap-southeast-2%2Fs3%2Faws4_request&X-Amz-Date=20240630T065727Z&X-Amz-Expires=300&X-Amz-SignedHeaders=host&X-Amz-Signature=e2d1420aa95d781924348806cd6427657ddd9183cc41a1f72f9066f63baeb864> | Parks, Gardens and Reserves directory  <https://www.nedlands.wa.gov.au/environment/parks-and-gardens/parks-ovals-and-reserves.aspx> |
| Peppermint Grove (S) | Local Planning Scheme - Zones and Reserves (DPLH-071)  Scheme zone for greenspace =   - Recreation - public | - | Reserves directory  <https://www.peppermintgrove.wa.gov.au/reserves.aspx> |
| City of Perth | Local Planning Scheme - Zones and Reserves (DPLH-071)  Scheme zone for greenspace =   - Parks and recreation | Open Space Framework  <https://engage.perth.wa.gov.au/open-space-framework> | - |
| City of Rockingham | Local Planning Scheme - Zones and Reserves (DPLH-071)  Scheme zone for greenspace =   - Public open space | Public open space community plan strategy  <https://nla.gov.au/nla.obj-2871529955/view> | Find a park or reserve  <https://rockingham.wa.gov.au/findavenue> |
| **Local Government** | **Spatial dataset/s** | **Public Open Space Strategy** | **LGA park / public open space listings** |
| Serpentine-Jarrahdale (S) | ocal Planning Scheme - Zones and Reserves (DPLH-071)  Scheme zone for greenspace =   - Public open space - Public purposes | Community Infrastructure And Public Open Space Strategy  <https://www.sjshire.wa.gov.au/Profiles/sj/Assets/ClientData/Document-Centre/Plans_and_Publications_/Community-Infrastructure-and-Public-Open-Space-Strategy.pdf> | Parks, reserves and playgrounds directory  <https://www.sjshire.wa.gov.au/community/your-community/parks-and-facilities/parks-reserves-and-playgrounds/parks-and-facilities.aspx> |
| City of South Perth | Local Planning Scheme - Zones and Reserves (DPLH-071)  Scheme zone for greenspace =   - Parks and recreation - Public purposes | Public Open Space Strategy  <https://southperth.wa.gov.au/docs/default-source/5-future/our-environment/water-management/public-open-space-strategy-part-2.pdf?sfvrsn=61fc0bd_0> | Parks and reserves directory  <https://southperth.wa.gov.au/discover/things-to-see-and-do/parks-reserves#:~:text=South%20Perth%20foreshore%20incorporates%20Sir,city%20across%20the%20Swan%20River>. |
| City of Stirling | Local Planning Scheme - Zones and Reserves (DPLH-071)  Scheme zone for greenspace =   - Public open space and local authority purposes | Public Open Space Strategy 2021 – 2031 Overview  <https://www.stirling.wa.gov.au/your-city/documents-and-publications/your-city/your-say/strategies-and-plans-(1)/public-open-space-strategy-summary> | Parks and playgrounds directory  <https://www.stirling.wa.gov.au/leisure-and-culture/attractions-and-recreation/parks-and-playgrounds> |
| City of Subiaco | Local Planning Scheme - Zones and Reserves (DPLH-071)  Scheme zone for greenspace =   - Public open space | Public Open Space Plan  <https://www.subiaco.wa.gov.au/CityofSubiaco/media/City-of-Subiaco/Your-council/Reports-and-corporate-documents/City-of-Subiaco-Public-Open-Space-Plan.pdf> | Parks & open spaces  <https://www.subiaco.wa.gov.au/see-do/places-to-visit/parks-open-spaces> |
| City of Swan | Local Planning Scheme - Zones and Reserves (DPLH-071)  Scheme zone for greenspace =   - Recreation - Public purposes - Special use | - | Parks, facilities and venues  <https://www.swan.wa.gov.au/explore-and-do/parks-venues-and-facilities> |
| Victoria Park (T) | Local Planning Scheme - Zones and Reserves (DPLH-071)  Scheme zone for greenspace =   - Parks and recreation | Public Open Space Strategy  <https://www.victoriapark.wa.gov.au/about/strategic-direction/strategic-programs/public-open-space-strategy> | Parks and reserves directory  <https://www.victoriapark.wa.gov.au/parks-and-reserves> |
| City of Vincent | Local Planning Scheme - Zones and Reserves (DPLH-071)  Scheme zone for greenspace =   - Public open space | Public Open Space Strategy  <https://imagine.vincent.wa.gov.au/public-open-space-strategy> | Parks and facilities directory  <https://www.vincent.wa.gov.au/parks-facilities> |
| City of Wanneroo | Local Planning Scheme - Zones and Reserves (DPLH-071)  Scheme zone for greenspace =   - Conservation - Parks and recreation |  | Parks directory  <https://www.wanneroo.wa.gov.au/parks> |
| **GREATER MELBOURNE** | | | |
| **Local Government** | **Spatial dataset/s** | **Public Open Space Strategy** | **LGA park / public open space listings** |
| Metropolitan Open Space Network | VEAC Metropolitan Melbourne Open Space Inventory.  The dataset consists of polygons delineating areas of public land(1) and local council land(2) that have been classified as public open space.  Source:  <https://discover.data.vic.gov.au/dataset/veac-metropolitan-melbourne-open-space-inventory> | <https://vpa.vic.gov.au/wp-content/uploads/2018/02/Open-Space-Network-Provision-and-Distribution-Reduced-Size.pdf>  <https://www.environment.vic.gov.au/__data/assets/pdf_file/0025/520594/Metro-Open-Space-Strategy-FA4-book-WEB.pdf> |  |
| City of Melbourne |  | City of Melbourne Open Space Strategy  Technical Report  <https://www.melbourne.vic.gov.au/SiteCollectionDocuments/open-space-strategy-technical-report.pdf> | Parks and public spaces  <https://www.melbourne.vic.gov.au/community/parks-open-spaces/Pages/parks-open-spaces.aspx> |
| Banyule City Council |  | Public Open Space Plan 2016-2031  <https://www.banyule.vic.gov.au/About-us/Policies-plans-strategies/Council-plans-and-strategies/Public-open-space-plan#:~:text=In%20addition%20public%20open%20space,physical%2C%20social%20and%20psychological%20development>. | Parks and reserves directory  <https://www.banyule.vic.gov.au/Events-activities/Parks-reserves> |
| Bayside City Council |  | Bayside Open Space Strategy 2012  <https://www.bayside.vic.gov.au/sites/default/files/2021-08/open_space_strategy_0.pdf> | Foreshores, parks and gardens  <https://www.bayside.vic.gov.au/where-are-our-parks-and-gardens> |
| City of Boroondara |  | Boroondara Open Space Strategy 2017  <https://www.boroondara.vic.gov.au/media/3481/download?inline=> | View all parks  <https://www.boroondara.vic.gov.au/recreation-arts/parks-and-gardens/find-park-near-you> |
| Brimbank City Council | Brimbank Parks and Open Spaces  This table provides the polygon boundaries of public accessible open space and categorised park reserves within Brimbank City Council.  <https://www.data.gov.au/data/dataset/brimbank-parks-and-open-spaces> | Creating Better Parks - Open Space and Playground Policy and Plan  <https://www.brimbank.vic.gov.au/about-council/how-we-work/policies-plans-and-strategies/plans/creating-better-parks-open-space-and-playground-policy-and-plan> | Parks and playgrounds directory  <https://www.brimbank.vic.gov.au/explore-brimbank/parks-and-nature/parks-and-playgrounds> |
| Cardinia Shire Council | Playgrounds within Cardinia Council  <https://www.data.gov.au/data/dataset/playgroundsincardinia> | Open Space Strategy  <https://creating.cardinia.vic.gov.au/open-space-strategy> | Parks, playgrounds and recreation reserves  <https://www.cardinia.vic.gov.au/parks> |
| **Local Government** | **Spatial dataset/s** | **Public Open Space Strategy** | **LGA park / public open space listings** |
| City of Casey | VEAC Metropolitan Melbourne Open Space Inventory.  <https://discover.data.vic.gov.au/dataset/veac-metropolitan-melbourne-open-space-inventory> | Open Space Strategy 2015  <https://www.casey.vic.gov.au/sites/default/files/2018-09/Open-Space-Strategy-Version-11-Sport-Leisure.pdf>  <https://www.casey.vic.gov.au/sites/default/files/2023-10/Open%20Space%20Strategy%20-%20City%20of%20Casey%20%28v2.0%29.pdf> | <https://www.casey.vic.gov.au/explore-casey/sports-facilities>  Parks and reserves directory  <https://www.casey.vic.gov.au/explore-casey/parks-reserves> |
| City of Darebin | VEAC Metropolitan Melbourne Open Space Inventory.  <https://discover.data.vic.gov.au/dataset/veac-metropolitan-melbourne-open-space-inventory> | Breathing Space: The Darebin Open Space Strategy  <https://www.yoursaydarebin.com.au/openspacestrategy> | https://www.darebin.vic.gov.au/Events-and-facilities/Parks  <https://www.darebin.vic.gov.au/Discover-Darebin/Spaces-and-places/Parks-and-Playgrounds> |
| Frankston City | Frankston City Council Playgrounds  <https://data.gov.au/dataset/ds-vic-6c5bb0bb-8f71-4a5f-ad0a-4271b8b094ca/details?q=frankston%20parks> | Open Space Strategy 2016-2036  <https://www.frankston.vic.gov.au/files/assets/public/our-community/community-development/pdfs/part_1_of_frankston_open_space_strategy_2016-2036_-_open_space_framework.pdf> | Parks and reserves directory  <https://www.frankston.vic.gov.au/Things-To-Do/Parks-and-Reserves?dlv_OC%20CL%20Public%20Parks%20Reserves%20Listing=(pageindex=1)> |
| City of Glen Eira | VEAC Metropolitan Melbourne Open Space Inventory.  <https://discover.data.vic.gov.au/dataset/veac-metropolitan-melbourne-open-space-inventory> | Open Space Strategy Refresh 2020  <https://www.gleneira.vic.gov.au/media/3427/open-space-strategy-refresh-2020.pdf> | Parks and playgrounds directory  <https://www.gleneira.vic.gov.au/our-city/parks-and-playgrounds?view=map>  <https://www.gleneira.vic.gov.au/our-city/parks-and-playgrounds> |
| Greater Dandenong | VEAC Metropolitan Melbourne Open Space Inventory.  <https://discover.data.vic.gov.au/dataset/veac-metropolitan-melbourne-open-space-inventory> | Open Space Strategy 2020-30  <https://www.greaterdandenong.vic.gov.au/open-space-strategy> | Parks and reserves directory  <https://www.greaterdandenong.vic.gov.au/open-spaces/parks-and-reserves?field_ra_category=All&facilities=All&field_playground_type_target_id=All&field=All&page=4> |
| City of Greater Geelong | Open Space - City of Greater Geelong  <https://data.gov.au/dataset/ds-dga-ccbaa516-31ef-4e86-b2b9-4e448fca24cc/details?q=geelong%20parks> | Social Infrastructure Plan Generation One: 2020-23  <https://www.geelongaustralia.com.au/common/Public/Documents/8d8353a5236fb5e-openspacenetworkreport-sipgenone2020-2023final.PDF> | Parks and reserves directory  <https://www.geelongaustralia.com.au/parks/default.aspx> |
| Hobsons Bay City Council | Parks and Open Spaces  Parks, reserves and open space areas in Hobsons Bay City Council.  <https://data.gov.au/dataset/ds-dga-211e3e82-b619-42ea-9d8f-c9b46437bf31/details?q=hobson%20bay%20parks> | Open Space Strategy <https://www.hobsonsbay.vic.gov.au/files/assets/public/documents/council/policies/open-space/hobsons-bay-open-space-strategy.pdf>  <https://www.hobsonsbay.vic.gov.au/Council/Strategy-Planning/Policies-Strategies-Plans/Open-Spaces> | Parks and reserves directory  <https://www.hobsonsbay.vic.gov.au/Community/Parks-Reserves> |
| **Local Government** | **Spatial dataset/s** | **Public Open Space Strategy** | **LGA park / public open space listings** |
| Hume City Council | VEAC Metropolitan Melbourne Open Space Inventory.  <https://discover.data.vic.gov.au/dataset/veac-metropolitan-melbourne-open-space-inventory> | Open Space Strategy <https://participate.hume.vic.gov.au/hume-open-space-strategy> | Parks and open space  <https://www.hume.vic.gov.au/Residents/Leisure-and-Open-Space/Parks-and-Reserves> |
| City of Kingston | VEAC Metropolitan Melbourne Open Space Inventory.  <https://discover.data.vic.gov.au/dataset/veac-metropolitan-melbourne-open-space-inventory> | Open Space Strategy  <https://www.kingston.vic.gov.au/council/council-documents/plans-policies-and-reports/open-space-strategy> | Parks and reserves directory  <https://www.kingston.vic.gov.au/Places-and-Events/Parks-and-Reserves> |
| Knox | VEAC Metropolitan Melbourne Open Space Inventory.  <https://discover.data.vic.gov.au/dataset/veac-metropolitan-melbourne-open-space-inventory> | Open Space Plan 2012-2022  <https://www.knox.vic.gov.au/sites/default/files/knox-files/our-council/policies-strategies-and-plans/knox-open-space-plan-sections1-3.pdf> | Parks and reserves directory  <https://www.knox.vic.gov.au/search?keywords=parks> |
| City of Manningham | Manningham Parks and Open Space  Spatial data showing the location of Parks and Open Space within Manningham City Council.  <https://data.gov.au/dataset/ds-dga-44a28224-9e9a-41c1-8168-488dfac1d831/details?q=manningham%20parks>  VEAC Metropolitan Melbourne Open Space Inventory.  <https://discover.data.vic.gov.au/dataset/veac-metropolitan-melbourne-open-space-inventory> | Open Space Strategy  <https://yoursay.manningham.vic.gov.au/openspacestrategy> | Parks and playspaces directory  <https://www.manningham.vic.gov.au/parks-and-playspaces-landing> |
| Maribyrnong City Council | VEAC Metropolitan Melbourne Open Space Inventory.  <https://discover.data.vic.gov.au/dataset/veac-metropolitan-melbourne-open-space-inventory> | Open Space Strategy  <https://www.maribyrnong.vic.gov.au/Building-planning/Current-and-future-planning/Strategies-and-policies/Open-Space-Strategy> | Parks directory  <https://www.maribyrnong.vic.gov.au/Park?dlv_OC%20CL%20Public%20Parks%20Reserves%20Listing=(pageindex=1)> |
| Maroondah City Council | VEAC Metropolitan Melbourne Open Space Inventory.  <https://discover.data.vic.gov.au/dataset/veac-metropolitan-melbourne-open-space-inventory> | Open Space Strategy  <https://www.maroondah.vic.gov.au/About-Council/Planning-for-our-future/Strategies-and-plans/Open-Space-Strategy-2016> | Parks and playgrounds directory  <https://www.maroondah.vic.gov.au/Explore/Parks-and-playgrounds> |
| City of Melbourne | VEAC Metropolitan Melbourne Open Space Inventory.  <https://discover.data.vic.gov.au/dataset/veac-metropolitan-melbourne-open-space-inventory> | Open space strategy  <https://www.melbourne.vic.gov.au/community/parks-open-spaces/policies-plans/Pages/open-space-strategy.aspx> | Parks and public spaces directory  <https://www.melbourne.vic.gov.au/community/parks-open-spaces/Pages/parks-open-spaces.aspx> |
| **Local Government** | **Spatial dataset/s** | **Public Open Space Strategy** | **LGA park / public open space listings** |
| Melton City Council | VEAC Metropolitan Melbourne Open Space Inventory.  <https://discover.data.vic.gov.au/dataset/veac-metropolitan-melbourne-open-space-inventory> | Open Space Plan 2016-2026 <https://www.melton.vic.gov.au/files/assets/public/council/publications/documentsreportsstrategies/plans/open-space-plan-background-report.pdf> | Parks & Recreation directory  <https://www.melton.vic.gov.au/Out-n-About/Leisure-Sport-Recreation/Parks-and-Trails/Parks-and-Reserves-in-Melton1/Parks-Recreation?dlv_OC%20CL%20Public%20Parks%20Reserves%20Listing=(pageindex=1)> |
| Mitchell Shire Council | VEAC Metropolitan Melbourne Open Space Inventory.  <https://discover.data.vic.gov.au/dataset/veac-metropolitan-melbourne-open-space-inventory>  Mitchell Shire Playgrounds  <https://data.gov.au/dataset/ds-dga-013ac8b9-dd2d-4d63-a482-abeae4b30282/details?q=mitchell%20parks> | Open Space Strategy 2013 – 2023  <https://cdn.mitchellshire.vic.gov.au/general-downloads/FINAL_MOSS_03OCT13_Web.pdf> | Parks, playgrounds and open space  <https://www.mitchellshire.vic.gov.au/our-region/parks-and-playgrounds?suburb=&keywords=#results> |
| City of Monash | VEAC Metropolitan Melbourne Open Space Inventory.  <https://discover.data.vic.gov.au/dataset/veac-metropolitan-melbourne-open-space-inventory> | Open Space Strategy  <https://www.monash.vic.gov.au/Planning-Development/Planning/Open-Space-Strategy> | Parks and recreation  <https://www.monash.vic.gov.au/Things-to-Do/Parks-Recreation> |
| City of Moonee Valley | VEAC Metropolitan Melbourne Open Space Inventory.  <https://discover.data.vic.gov.au/dataset/veac-metropolitan-melbourne-open-space-inventory>  Moonee Valley – Playground  <https://data.gov.au/dataset/ds-dga-be20e0d5-67b4-4803-997e-0ef90635e308/details?q=moonee%20valley%20parks> | Open space strategy  <https://yoursay.mvcc.vic.gov.au/openspace> | Open spaces and leisure places  <https://mvcc.vic.gov.au/play/my-outdoors/sports-parks-playgrounds-pools/#open-spaces> |
| Merri-bek | VEAC Metropolitan Melbourne Open Space Inventory.  <https://discover.data.vic.gov.au/dataset/veac-metropolitan-melbourne-open-space-inventory> | Open space strategy  <https://hdp-au-prod-app-more-conversations-files.s3.ap-southeast-2.amazonaws.com/9817/0198/1109/Draft_Open_Space_Strategy_-_December_2023.pdf> | Parks and gardens  <https://www.merri-bek.vic.gov.au/exploring-merri-bek/parks-and-gardens/> |
| Mornington Peninsula Shire | VEAC Metropolitan Melbourne Open Space Inventory.  <https://discover.data.vic.gov.au/dataset/veac-metropolitan-melbourne-open-space-inventory> | Open space strategy  <https://www.mornpen.vic.gov.au/files/assets/public/new-website-documents/about-us/strategies-amp-plans/docs/open_space_reportvol1_final_june_03_pdf.pdf> | Parks and reserves directory  <https://www.mornpen.vic.gov.au/Activities/Parks-Reserves> |
| Nillumbik Shire | VEAC Metropolitan Melbourne Open Space Inventory.  <https://discover.data.vic.gov.au/dataset/veac-metropolitan-melbourne-open-space-inventory> | Open space strategy  <https://www.nillumbik.vic.gov.au/files/assets/public/council/council-publications/strategies-etc/open-space-strategy-nov-15-2005.pdf> | Parks and playgrounds  <https://www.nillumbik.vic.gov.au/Living-in/Parks-and-playgrounds> |
| **Local Government** | **Spatial dataset/s** | **Public Open Space Strategy** | **LGA park / public open space listings** |
| City of Port Phillip | VEAC Metropolitan Melbourne Open Space Inventory.  <https://discover.data.vic.gov.au/dataset/veac-metropolitan-melbourne-open-space-inventory> | Places for People: Public Space Strategy  <https://www.portphillip.vic.gov.au/about-the-council/strategies-policies-and-plans/places-for-people-public-space-strategy> | Parks and playgrounds  <https://www.portphillip.vic.gov.au/explore-the-city/beaches-parks-and-playgrounds/find-parks-and-playgrounds> |
| Borough of Queenscliffe | VEAC Metropolitan Melbourne Open Space Inventory.  <https://discover.data.vic.gov.au/dataset/veac-metropolitan-melbourne-open-space-inventory> | - | Parks, ovals and playgrounds  <https://www.queenscliffe.vic.gov.au/Things-to-do/Parks-and-public-spaces/Parks-ovals-and-playgrounds> |
| City of Stonnington | VEAC Metropolitan Melbourne Open Space Inventory.  <https://discover.data.vic.gov.au/dataset/veac-metropolitan-melbourne-open-space-inventory> | Open spaces strategy  <https://www.stonnington.vic.gov.au/Planning-and-building/Strategic-planning/Planning-Strategies/Open-Spaces-Strategy> | Parks, reserves and gardens  <https://www.stonnington.vic.gov.au/Whats-On/Explore-our-parks-trails-and-heritage/Parks-reserves-and-gardens> |
| City of Whitehorse | VEAC Metropolitan Melbourne Open Space Inventory.  <https://discover.data.vic.gov.au/dataset/veac-metropolitan-melbourne-open-space-inventory> | Open space strategy  <https://www.whitehorse.vic.gov.au/sites/whitehorse.vic.gov.au/files/assets/documents/Open-Space-Strategy-Part-1.pdf> | Parks and bushland reserves  <https://www.whitehorse.vic.gov.au/things-do/parks-playgrounds/parks-and-bushland-reserves> |
| City of Whittlesea | VEAC Metropolitan Melbourne Open Space Inventory.  <https://discover.data.vic.gov.au/dataset/veac-metropolitan-melbourne-open-space-inventory> | Open space strategy  <https://www.whittlesea.vic.gov.au/media/qp1poqe0/1-open-space-strategy-summary-report.pdf> | Parks and playgrounds  <https://www.whittlesea.vic.gov.au/arts-events-recreation/parks-and-playgrounds/> |
| Wyndham | VEAC Metropolitan Melbourne Open Space Inventory.  <https://discover.data.vic.gov.au/dataset/veac-metropolitan-melbourne-open-space-inventory> | Open space strategy 2045  <https://www.wyndham.vic.gov.au/sites/default/files/2016-06/Wyndham%20Open%20Space%20Strategy%20-%20WOSS%20FINAL%20-%20MAIN.pdf> | Sports, parks and recreation  <https://www.wyndham.vic.gov.au/services/sports-parks-recreation> |
| City of Yarra | VEAC Metropolitan Melbourne Open Space Inventory.  <https://discover.data.vic.gov.au/dataset/veac-metropolitan-melbourne-open-space-inventory> | Open space strategy 2020  <https://www.yarracity.vic.gov.au/-/media/files/ycc/about-us/strategies/yoss2020summaryreportfinal150920.pdf?la=en> | Parks and reserves  <https://www.yarracity.vic.gov.au/facilities/?type=Parks-and-gardens> |
| Yarra Ranges | VEAC Metropolitan Melbourne Open Space Inventory.  <https://discover.data.vic.gov.au/dataset/veac-metropolitan-melbourne-open-space-inventory> | Recreation and open space strategy  <https://www.yarraranges.vic.gov.au/Council/Corporate-documents/Policies-strategies/Recreation-and-open-space-strategy> | Parks & recreation  <https://www.yarraranges.vic.gov.au/Experience/Parks-Recreation> |
| **GREATER SYDNEY** | | | |
| **Local Government** | **Spatial dataset/s** | **Public Open Space Strategy** | **LGA park / public open space listings** |
| Sydney Green Grid | Sydney green grid / Green Assets spatial data layer obtained from the Department of Planning, Industry and Environment.  Environmental Planning Instrument - Land Zoning: Identifies land use zones and the type of land uses that are permitted (with or without consent) or prohibited in each zone on any given land as designated by the relevant NSW environmental planning instrument (EPI) under the Environmental Planning and Assessment Act 1979.  Source: <https://www.planningportal.nsw.gov.au/opendata/dataset/environment-planning-instrument-local-environmental-plan-land-zoning> **Wales** | <https://www.governmentarchitect.nsw.gov.au/projects/sydney-green-grid> | - |
| Bayside Council | Department of Planning, Industry and Environment Sydney green grid / Green Assets spatial data layer | Open Space Strategy  <https://www.bayside.vic.gov.au/sites/default/files/2021-08/open_space_strategy_0.pdf> | Parks  <https://www.bayside.nsw.gov.au/area/parks> |
| City of Blacktown | Department of Planning, Industry and Environment Sydney green grid / Green Assets spatial data layer | Recreation and Open Space Strategy  <https://www.blacktown.nsw.gov.au/files/assets/public/blacktown-city-council-ross.pdf> | Parks and recreation directory  <https://www.blacktown.nsw.gov.au/Sport-and-recreation/Parks-and-recreation-directory> |
| Blue Mountains City Council | Department of Planning, Industry and Environment Sydney green grid / Green Assets spatial data layer | Open Space and Recreation Strategic Plan  <https://www.bmcc.nsw.gov.au/sites/default/files/docs/Open-Space-Recreation-Strategic-Plan.pdf> | Parks, sportsgrounds & sport courts  <https://www.bmcc.nsw.gov.au/parks> |
| Burwood Council | Department of Planning, Industry and Environment Sydney green grid / Green Assets spatial data layer | Community Facilities and Open Space Strategy  <https://www.burwood.nsw.gov.au/files/sharedassets/public/have-your-say/community-facilities-and-open-space-strategy.pdf> | Parks and reserves  <https://www.burwood.nsw.gov.au/For-Residents/Parks-and-Recreation/Parks-and-Reserves> |
| Camden Council | Department of Planning, Industry and Environment Sydney green grid / Green Assets spatial data layer | Draft spaces and places strategy 2020  <https://www.camden.nsw.gov.au/assets/Uploads/20-297647-66467-Camden-Council-Draft-Spaces-and-Places-Strategy-2020-distributed-version-15-9-2020.pdf> | Halls, parks and playgrounds  <https://www.camden.nsw.gov.au/parks-and-recreation/parks-and-playgrounds/> |
| Campbelltown City Council | Department of Planning, Industry and Environment Sydney green grid / Green Assets spatial data layer | Open space directions and strategies report  <https://www.campbelltown.sa.gov.au/__data/assets/pdf_file/0023/234239/Open-Space-Strategy.pdf> | Look for a park/sportsground or things to do  <https://www.campbelltown.nsw.gov.au/ParksSportandRecreation/RLFPS/LookForParkSportsground?dlv_LV%20Public%20Park%20and%20Recreation=(pageindex=1)> |
| **Local Government** | **Spatial dataset/s** | **Public Open Space Strategy** | **LGA park / public open space listings** |
| City of Canada Bay | Department of Planning, Industry and Environment Sydney green grid / Green Assets spatial data layer | Social Infrastructure (Open Space and Recreation) Strategy and Action Plan  <https://hdp-au-prod-app-cbay-collaborate-files.s3.ap-southeast-2.amazonaws.com/2215/7187/9519/Canada_Bay_Recreation_and_Open_Space_Strategy.pdf> | Parklands and waterways  <https://www.canadabay.nsw.gov.au/lifestyle/parklands-and-waterways/parks> |
| Canterbury-Bankstown | Department of Planning, Industry and Environment Sydney green grid / Green Assets spatial data layer | Open space strategic plan  <https://www.cbcity.nsw.gov.au/council/planning-for-the-city/open-space-planning> | Parks and sporting fields  <https://www.cbcity.nsw.gov.au/environment/parks-reserves> |
| Cumberland City Council | Department of Planning, Industry and Environment Sydney green grid / Green Assets spatial data layer | Open space and recreation strategy  <https://www.cumberland.nsw.gov.au/sites/default/files/inline-files/open-space-recreation-strategy-2019-2029.pdf> | Park and gardens  <https://www.cumberland.nsw.gov.au/parks-and-gardens> |
| Fairfield City | Department of Planning, Industry and Environment Sydney green grid / Green Assets spatial data layer | Open space strategy  <https://www.fairfieldcity.nsw.gov.au/files/assets/public/documents/plan_build/openspace_strategy_report.pdf> | Parks, sportsfields and public spaces  <https://www.fairfieldcity.nsw.gov.au/Services/Sports-Parks-Recreation/Find-a-Sportsfield?dlv_OC%20CL%20Public%20Parks%20Reserves%20Listing=(pageindex=1)> |
| Georges River Council | Department of Planning, Industry and Environment Sydney green grid / Green Assets spatial data layer | Open Space Recreation and Community Facilities Strategy  <https://www.georgesriver.nsw.gov.au/StGeorge/media/Documents/Council/Publications/Open-Space-Recreation-and-Community-Facilities-Strategy.pdf> | Parks and reserves  <https://www.georgesriver.nsw.gov.au/Environment/Parks-and-Reserves> |
| Hawkesbury City Council | Department of Planning, Industry and Environment Sydney green grid / Green Assets spatial data layer | Draft open space strategy  <https://www.hawkesbury.nsw.gov.au/__data/assets/pdf_file/0004/53590/ORD_FEB05_2013_Att2toItem13.pdf> | Love your Hawkesbury parklands  <https://www.hawkesbury.nsw.gov.au/__data/assets/pdf_file/0003/155226/Love-your-Hawkesbury-Parklands-Brochure.pdf> |
| Hornsby Shire Council | Department of Planning, Industry and Environment Sydney green grid / Green Assets spatial data layer | Active living strategy  <https://www.hornsby.nsw.gov.au/__data/assets/pdf_file/0005/74444/Active-Living-Hornsby-Strategy-Final-Draft-Report-low-res.pdf> | Parks and playgrounds  <https://www.hornsby.nsw.gov.au/lifestyle/sports-and-recreation/parks-and-playgrounds> |
| Hunters Hill Council | Department of Planning, Industry and Environment Sydney green grid / Green Assets spatial data layer | - | Parks and reserves  <https://www.huntershill.nsw.gov.au/Recreation/Parks-and-Reserves> |
| Inner West | Department of Planning, Industry and Environment Sydney green grid / Green Assets spatial data layer | Recreation strategy and action plan  <https://yoursay.innerwest.nsw.gov.au/recreation-strategy-and-action-plan-2023> | Parks by facility  <https://www.innerwest.nsw.gov.au/explore/parks-sport-and-recreation/parks-and-playgrounds/parks-by-facility> |
| **Local Government** | **Spatial dataset/s** | **Public Open Space Strategy** | **LGA park / public open space listings** |
| Ku-ring-gai Council | Department of Planning, Industry and Environment Sydney green grid / Green Assets spatial data layer | Ku-ring-gai Open Space Acquisition Strategy 2006  <https://www.krg.nsw.gov.au/Planning-and-development/Planning-policies-and-guidelines/Strategies-and-management-plans/Open-space-acquisition-program> | Parks, playgrounds and sportsfields  <https://www.krg.nsw.gov.au/Things-to-do/Parks-playgrounds-and-sportsfields?dlv_OC%20CL%20Public%20Parks%20Reserves%20Listing=(pageindex=1)> |
| Lane Cove Council | Department of Planning, Industry and Environment Sydney green grid / Green Assets spatial data layer | Open space plan  <http://www.lanecove.nsw.gov.au/YourCouncil/CouncilsPlans/Pages/OpenSpacePlans.aspx> | Parks and recreation  <http://www.lanecove.nsw.gov.au/Community/ParksandRecreation/Pages/Parks.aspx> |
| Liverpool City Council | Department of Planning, Industry and Environment Sydney green grid / Green Assets spatial data layer | Recreation, open space And sports strategy  <https://www.liverpool.nsw.gov.au/trim/documents?RecordNumber=350490.2018> | Open space  <https://www.liverpool.nsw.gov.au/venues/parks-and-playgrounds/open-space> |
| Mosman Council | Department of Planning, Industry and Environment Sydney green grid / Green Assets spatial data layer | Open Space Recreational Needs Assessment  <https://mosman.nsw.gov.au/sites/default/files/2021-04/Open%20Space%20Recreational%20Needs%20Asessment%20-%20Adopted%206%20Oct%202015.pdf> | Parks and reserves  <https://mosman.nsw.gov.au/recreation/parks-and-reserves> |
| North Sydney Council | Department of Planning, Industry and Environment Sydney green grid / Green Assets spatial data layer | Open space provision strategy  <https://www.northsydney.nsw.gov.au/directory-record/22885/open-space-provision-strategy> | Parks and reserves  <https://www.northsydney.nsw.gov.au/Recreation_Facilities/Parks_Reserves/Parks_Reserves_Management> |
| Northern Beaches Council | Department of Planning, Industry and Environment Sydney green grid / Green Assets spatial data layer | Let's Play! Open Space and Outdoor Recreation Strategy and Action Plan  <https://yoursay.northernbeaches.nsw.gov.au/open-space-and-recreation-strategy> | Parks and Reserves list  <https://www.northernbeaches.nsw.gov.au/things-to-do/parks-and-trails/parks-and-reserves> |
| City of Parramatta | Department of Planning, Industry and Environment Sydney green grid / Green Assets spatial data layer | Draft social infrastructure strategy: part 2 – recreation and open space  <https://www.cityofparramatta.nsw.gov.au/sites/council/files/2017-08/Draft%20Social%20Infrastructure%20Strategy%20-%20Section%202.pdf> | List of parks and reserves  <https://www.cityofparramatta.nsw.gov.au/recreation/our-parks/list-of-parks-and-reserves> |
| Penrith City Council | Department of Planning, Industry and Environment Sydney green grid / Green Assets spatial data layer | Sport and recreation strategy  <https://www.penrithcity.nsw.gov.au/images/sport__rec_policy_a4_strategy_mar2020_v2_access.pdf> | Outdoor recreation  <https://www.penrithcity.nsw.gov.au/facilities-recreation/outdoor-recreation> |
| **Local Government** | **Spatial dataset/s** | **Public Open Space Strategy** | **LGA park / public open space listings** |
| Randwick City Council | Department of Planning, Industry and Environment Sydney green grid / Green Assets spatial data layer | Open space and recreation strategy  <https://ehq-production-australia.s3.ap-southeast-2.amazonaws.com/2879d6af23af04130f86ab10e18abcfc8e2f7051/original/1622112825/0ffe968b787eb5b66e0633156f7bdeaf_Draft_Open_Space_and_Recreation_Strategy.pdf?X-Amz-Algorithm=AWS4-HMAC-SHA256&X-Amz-Credential=AKIAIBJCUKKD4ZO4WUUA%2F20210810%2Fap-southeast-2%2Fs3%2Faws4_request&X-Amz-Date=20210810T135541Z&X-Amz-Expires=300&X-Amz-SignedHeaders=host&X-Amz-Signature=e0017d62114072ae9fb2cb5bf39eaa4a7d7cb0f640fc92c842972321388dacb9> | Parks  <https://www.randwick.nsw.gov.au/facilities-and-recreation/parks> |
| City of Ryde | Department of Planning, Industry and Environment Sydney green grid / Green Assets spatial data layer | Integrated open space plan  <https://www.ryde.nsw.gov.au/files/assets/public/publications/parks-open-space/integrated-open-space-plan-2012.pdf> | Find a park or sportsground  <https://www.ryde.nsw.gov.au/Recreation/Parks-and-Sportsgrounds/Find-a-Park-or-Sportsground?dlv_LV%20Public%20Park=(pageindex=1)> |
| Strathfield Council | Department of Planning, Industry and Environment Sydney green grid / Green Assets spatial data layer | - | Parks and natural areas  <https://www.strathfield.nsw.gov.au/play/parks-and-natural-areas/> |
| Sutherland Shire | Department of Planning, Industry and Environment Sydney green grid / Green Assets spatial data layer | Open space strategy and implementation plan 2021 – 2031  <https://www.sutherlandshire.nsw.gov.au/__data/assets/pdf_file/0016/11716/2021-Open-Space-Strategy-and-Implementation-Plan-Press-A7848255.pdf> | Parks and reserves  <https://www.sutherlandshire.nsw.gov.au/Outdoors/Parks-and-Playgrounds> |
| City of Sydney | Department of Planning, Industry and Environment Sydney green grid / Green Assets spatial data layer | Public spaces strategies  <https://www.cityofsydney.nsw.gov.au/built-environment/public-spaces> | Parks and open spaces  <https://www.cityofsydney.nsw.gov.au/parks> |
| The Hills Shire | Department of Planning, Industry and Environment Sydney green grid / Green Assets spatial data layer | Recreation strategy  <https://www.thehills.nsw.gov.au/files/sharedassets/public/ecm-website-documents/page-documents/building/plans-guidelines/recreation_strategy.pdf> | Parks and recreation directory  <https://www.thehills.nsw.gov.au/Venues/Parks-Recreation/Parks-Recreation-Directory?dlv_OC%20CL%20Public%20Parks%20Reserves%20Listing=(pageindex=1)> |
| Waverley Council | Department of Planning, Industry and Environment Sydney green grid / Green Assets spatial data layer | Open space and recreation strategy  <https://www.waverley.nsw.gov.au/recreation/parks/open_space_and_recreation_strategy> | Parks  <https://www.waverley.nsw.gov.au/recreation/parks> |
| **Local Government** | **Spatial dataset/s** | **Public Open Space Strategy** | **LGA park / public open space listings** |
| City of Willoughby | Department of Planning, Industry and Environment Sydney green grid / Green Assets spatial data layer | Public spaces and recreation strategy  <https://ehq-production-australia.s3.ap-southeast-2.amazonaws.com/6712d5875bfed4fc97c0389114f194968d5f7061/original/1711580154/cb252993e61c879868a1a376f3a6c6a4_Public_Spaces_and_Recreation_Strategy.pdf?X-Amz-Algorithm=AWS4-HMAC-SHA256&X-Amz-Credential=AKIA4KKNQAKIOR7VAOP4%2F20240705%2Fap-southeast-2%2Fs3%2Faws4_request&X-Amz-Date=20240705T022137Z&X-Amz-Expires=300&X-Amz-SignedHeaders=host&X-Amz-Signature=a64f79c9a60a8ad204df871459a65657547cf399d4e505cba0e7deb526262c3d> | Parks, reserves and playgrounds  <https://www.willoughby.nsw.gov.au/Residents/Parks-and-recreation/Parks-reserves-and-playgrounds> |
| Woollahra Municipal Council | Department of Planning, Industry and Environment Sydney green grid / Green Assets spatial data layer | Open Space & Recreation Strategies  <https://www.woollahra.nsw.gov.au/Recreation/Open-Space-Recreation-Strategies-And-Plans-Of-Management> | Parks, reserves and playgrounds  <https://www.woollahra.nsw.gov.au/recreation/parks,_reserves_and_playgrounds/list_of_parks_and_playgrounds> |
| **GREATER HOBART** | | | |
| **Local Government** | **Spatial dataset/s** | **Public Open Space Strategy** | **LGA park / public open space listings** |
| Tasmanian open space strategy | The LIST Local Government Reserves are those areas created by subdivision under the Local Government Act.  Source: <https://www.thelist.tas.gov.au/app/content/data/geo-meta-data-record?detailRecordUID=5cff8723-56ce-4ce7-ad7a-c8245fbfc31e>  The LIST Public Land Classification dataset is the authoritative source for information on Tasmania's Crown Land Reserves.  Source: <https://www.thelist.tas.gov.au/app/content/data/geo-meta-data-record?detailRecordUID=d924bf33-9150-46a1-b339-b350efa8332c#:~:text=The%20LIST%20Public%20Land%20Classification,the%20Crown%20Lands%20Act%201976> | The Tasmanian Open Space Policy and Planning Framework | - |
| City of Hobart | Open Space Parks  <https://data.gov.au/dataset/ds-hobart-https%3A%2F%2Fwww.arcgis.com%2Fhome%2Fitem.html%3Fid%3D773ad2ecf1304a5fabe9a5e580c11586%26sublayer%3D0/details?q=hobart%20parks>  City of Hobart Playground Locations  <https://data.gov.au/dataset/ds-hobart-https%3A%2F%2Fwww.arcgis.com%2Fhome%2Fitem.html%3Fid%3D0493412ed1b54a96ae23bb6a76fc2f93%26sublayer%3D0/details?q=hobart%20parks> | Open space in Hobart  <https://ehq-production-australia.s3.ap-southeast-2.amazonaws.com/830855fe8690ff60eb8e62a15e163973aad550e6/original/1680253028/3cef328022ef249dc998f871787e0b91_Central_Hobart_Open_Space_FINAL_17-02-2023.pdf?X-Amz-Algorithm=AWS4-HMAC-SHA256&X-Amz-Credential=AKIA4KKNQAKIOR7VAOP4%2F20240705%2Fap-southeast-2%2Fs3%2Faws4_request&X-Amz-Date=20240705T022836Z&X-Amz-Expires=300&X-Amz-SignedHeaders=host&X-Amz-Signature=46f5e0434096dc287e9f61c03764bd441e629afcce1c0b304489cb3f18659f00> | Parks, sportsgrounds and reserves  <https://www.hobartcity.com.au/Community/Parks-reserves-and-sporting-facilities> |
| **AUSTRALIAN CAPITAL TERRITORY** | | | |
| **Local Government** | **Spatial dataset/s** | **Public Open Space Strategy** | **LGA park / public open space listings** |
| Canberra | ACT Government open space dataset.  Source: <https://actmapi-actgov.opendata.arcgis.com/datasets/d2711228d67e41b5a4ae76fb528292b3_0/explore?location=-35.361489%2C149.047230%2C11.53>  ACTGOV Park Feature Assets  <https://data.gov.au/dataset/ds-actmapi-https%3A%2F%2Fwww.arcgis.com%2Fhome%2Fitem.html%3Fid%3D2f01b82000fb40858b2f186feb271fac%26sublayer%3D1/details?q=palmerston%20parks> | National Capital Open Space System (NCOSS) Review  [https://www.nca.gov.au/planning/plans-policies-and-guidelines/planning-policy-review/national-capital-open-space-system#](https://www.nca.gov.au/planning/plans-policies-and-guidelines/planning-policy-review/national-capital-open-space-system) | Parks ACT  <https://www.environment.act.gov.au/parks-conservation/parks-and-reserves/find-a-park> |
| **GREATER BRISBANE** | | | |
| **Local Government** | **Spatial dataset/s** | **Public Open Space Strategy** | **LGA park / public open space listings** |
| Brisbane City Council | Park Locations: a spatial dataset that contains the boundary, address, and a point location for each park.  Source: <https://www.data.brisbane.qld.gov.au/data/dataset/park-locations>CT | - | Parks  <https://www.brisbane.qld.gov.au/things-to-see-and-do/council-venues-and-precincts/parks> |
| City of Moreton Bay | - | Open space strategy  <https://www.moretonbay.qld.gov.au/files/assets/public/v/1/services/publications/planning-strategies/open-space-strategy.pdf> | Sporting facilities, parks and playgrounds  <https://www.moretonbay.qld.gov.au/Services/Sport-Recreation/Parks> |
| Ipswich | Ipswich Parks and Reserves  <https://data.gov.au/dataset/ds-dga-32cdcdbf-e454-4ad8-80fd-02edc1662a44/details?q=ipswich%20parks> | Open space and recreation strategy  <https://www.ipswich.qld.gov.au/__data/assets/pdf_file/0017/112544/ICC-Open-Space-and-Recreation-Strategy-2019.pdf> | Parks search  <https://www.ipswich.qld.gov.au/explore/parks_reserves_precincts/parks_search> |
| City of Gold Coast | Parks Playing Surfaces  <https://data.gov.au/dataset/ds-dga-07d7fc2c-d923-4d10-bf39-edd0c5c8b450/details?q=gold%20coast%20parks> | - | Park finder  <https://www.goldcoast.qld.gov.au/Things-to-do/Parks-gardens-reserves/Park-Finder> |
| Sunshine Coast Council | - | Open space strategy  <https://els.sunshinecoast.qld.gov.au/explore-by-theme/open-space> | Parks and reserves  <https://www.sunshinecoast.qld.gov.au/environment/education-resources-and-events/environment-resources-and-publications/parks-and-reserves> |
| **GREATER DARWIN** | | | |
| **Local Government** | **Spatial dataset/s** | **Public Open Space Strategy** | **LGA park / public open space listings** |
| City of Darwin | Land zoned in the City of Darwin as parks.  Source: <https://open-darwin.opendata.arcgis.com/maps/614c63f438cb48269b83a9fdd41b939b/about> | Darwin Inner Suburbs area plan  <https://planinc.org.au/images/stories/documents/Darwin%20Inner%20Suburbs%20Area%20PLan%20Ammendment%20440.pdf>  Play space strategy  <https://www.darwin.nt.gov.au/council/about-council/publications-and-forms/play-space-strategy> | Parks and playgrounds  <https://www.darwin.nt.gov.au/explore/facilities/parks-playgrounds/overview> |
| City of Palmerstone | - | - | Parks and playgrounds  <https://www.palmerston.nt.gov.au/live/facilities-recreation/recreation/parks-and-playgrounds> |
